# Editorial: Managing cancer metastasis by tackling anticancer drug resistance

**DOI:** 10.3389/fphar.2024.1432248

**Published:** 2024-06-10

**Authors:** Erxi Wu, Zhongzhi Wu, Chao-Yie Yang, Dan Qi, Xiaoxiao Hu, Wei Li

**Affiliations:** ^1^ Department of Neurosurgery and Neuroscience Institute, Baylor Scott & White Health, Temple, TX, United States; ^2^ Department of Neurosurgery, Baylor College of Medicine, Temple, TX, United States; ^3^ School of Medicine, Texas A&M University, College Station, TX, United States; ^4^ Irma Lerma Rangel School of Pharmacy, Texas A&M University, College Station, TX, United States; ^5^ LIVESTRONG Cancer Institutes and Department of Oncology, Dell Medical School, The University of Texas at Austin, Austin, TX, United States; ^6^ Department of Pharmaceutical Sciences, College of Pharmacy, University of Tennessee Health Science Center, Memphis, TN, United States; ^7^ State Key Laboratory of Chemo/Biosensing and Chemometrics, College of Biology, Molecular Science and Biomedicine Laboratory and Aptamer Engineering Center of Hunan Province, Hunan University, Changsha, China; ^8^ Shenzhen Research Institute, Hunan University, Shenzhen, China; ^9^ Innovation Institute of Industrial Design and Machine Intelligence, Quanzhou-Hunan University, Quanzhou, China; ^10^ Research Institute of Hunan University in Chongqing, Chongqing, China

**Keywords:** drug resistance, metastasis, cancer treatment, anti-cancer agents, resistance mechanisms, therapeutic strategies

## Introduction

“I think the next Frontier in precision genomic medicine is figuring out how to circumvent resistance,” said Laurie H. Glimcher, M.D., president and CEO of Dana-Farber Cancer Institute, at a lecture at the National Institutes of Health on 7 November 2016 (National Cancer Institute, Why Do Cancer Treatments Stop Working? Overcoming Treatment Resistance, 2016). Drug resistance and tumor metastasis remain formidable obstacles in the effective treatment of cancer, contributing to treatment failure and poor patient outcomes (Chaffer and Weinberg, 2011; Gao et al., 2014; Gottesman et al., 2016; Steeg, 2016; Lambert et al., 2017; Fares et al., 2020; Yang et al., 2020; Nussinov et al., 2021; Labrie et al., 2022). Understanding the underlying mechanisms driving drug resistance and metastatic spread is crucial for devising novel therapeutic strategies to combat these challenges. This Research Topic seeks to compile recent advancements in elucidating drug resistance mechanisms associated with metastasis and cancer progression, while also exploring innovative approaches to develop new anti-cancer agents capable of overcoming these obstacles. Contributions are welcomed on Research Topic ranging from novel anti-cancer strategies and mechanisms of drug resistance and metastasis to innovative approaches aimed at enhancing therapeutic efficacy against metastatic cancers.

## Unraveling drug resistance in metastatic colorectal cancer: key pathways

Currently, colorectal cancer ranks as the third most common and deadliest cancer globally, with 20% of patients presenting with metastatic CRC (mCRC) at diagnosis and another 25% developing metastases later (Biller and Schrag, 2021). Despite advances in treatment modalities such as chemotherapy and targeted therapies, the prognosis of patients with mCRC remains poor with a 5-year survival rate of only 14%, largely due to therapeutic resistance. Albadari et al. highlights the major resistance mechanisms in mCRC, particularly focusing on drug transporters, EGFR mutations, and the HGF/c-MET signaling pathway, which promote tumor invasion and metastasis, and discusses emerging strategies to overcome these challenges.

## Advances and challenges in ovarian cancer treatment: A decade of research on drug resistance and poly ADP-Ribose polymerase inhibitors

Liu et al. analyzes the trends in ovarian cancer and chemotherapy resistance research from 2013 to 2022 using bibliometric software such as CiteSpace and VosViewer, examining data from the Web of Science Core Research Topic. There is an increasing focus on understanding the mechanisms underlying drug resistance and the clinical efficacy of poly ADP-ribose polymerase inhibitors (PARPis) and the antiangiogenic agent bevacizumab, with significant contributions from Chinese researchers and institutions. The findings highlight ongoing challenges in overcoming drug resistance, suggesting a need for innovative treatment strategies and further exploration of resistance mechanisms.

Furthermore, Dong et al. discusses the increasing incidence of ovarian cancer in China and the clinical use of PARPis that target DNA repair enzymes to treat tumors with homologous recombination dysfunction, particularly in advanced ovarian epithelial cancer. This review highlights the growing challenge of intrinsic or acquired PARPi resistance and summarizes current advances in combination strategies to enhance PARPi efficacy.

## Advancements in small molecule therapies targeting estrogen receptors in breast cancer

The estrogen receptor (ER) is integral to multiple signaling pathways. Its targeting therapies, including selective estrogen receptor modulators (SERMs) such as tamoxifen, play pivotal roles in inhibiting breast cancer cell proliferation, among other benefits. However, challenges such as drug resistance and side effects on tissues expressing high levels of ER such as the endometrium complicate their clinical use. Yao et al. discusses recent developments in novel ER-targeted drugs, specifically selective estrogen receptor degraders (SERDs) and covalent antagonists (SERCAs), focusing on their optimization and potential in treating breast cancer and other ER-related conditions.

## Identification of new benzylquinazoline compounds as inhibitors of p97/VCP

In a study by Zhang et al., novel DBeQ analogs were synthesized and evaluated as potential inhibitors of the ATPase valosin-containing protein (VCP/p97). Compounds **6** and **7** in this report exhibited higher potency than known inhibitors, inducing cell cycle arrest and modulating key signaling pathways. Moreover, compound **6** demonstrated promising antitumor effects and reduced toxicity *in vivo*, suggesting its potential as a selective and effective p97 inhibitor with improved clinical prospects.

## SAF-189s potently inhibits lorlatinib-resistant NSCLC with ALK mutations L1196M and D1203N

Li et al. present a clinical case of metastatic ALK-rearranged NSCLC developing rare, acquired compound ALK mutations (L1196M and D1203N) conferring resistance to lorlatinib. The patient showed stable and controlled disease progression with SAF-189s treatment for 3 months, followed by a partial response to chemotherapy (pemetrexed-carboplatin) with bevacizumab, maintained for 7 months. This study offered valuable insights, suggesting that combination of SAF-189s and chemotherapy may be potential therapeutic options for NSCLC patients with ALK L1196M/D1203N compound mutations.

## Olverembatinib monotherapy: a chemo-free option for elderly patients with relapsed Ph-positive ALL

The introduction of BCR/ABL1 tyrosine kinase inhibitors (TKIs) has significantly improved outcomes for Philadelphia chromosome-positive acute lymphoblastic leukemia (Ph^+^ -ALL) patients, but relapse remains common due to acquired drug resistance. Third generation TKIs such as olverembatinib are promising solutions. In this case report by Ding and Li, a 79-year-old female patient with relapsed Ph^+^ -B-ALL, despite frailty, achieved a second complete molecular remission (2nd CMR) with olverembatinib monotherapy, suggesting its potential as a safe and effective chemo-free option for elderly Ph^+^-ALL patients experiencing relapse after prior therapy.

## Perspectives

In summary, this Research Topic aimed to advance our understanding of drug resistance mechanisms, to showcase the development of novel anti-cancer agents, and clinical examples of combination therapy and new generation of targeted therapy to combat metastases. With the collaborative effort contributed to this Research Topic, we strive to make meaningful progress in overcoming drug resistance, alleviating the burden of metastatic cancers and improving patient outcomes.

We extend our sincere appreciation to the authors for their valuable contributions, and we express our gratitude to the reviewers for their expert evaluations and generous dedication of time and efforts.
